# The role of insulin sensitivity and intranasally applied insulin on olfactory perception

**DOI:** 10.1038/s41598-019-43693-7

**Published:** 2019-05-10

**Authors:** Sharmili Edwin Thanarajah, Vera Hoffstall, Lionel Rigoux, Ruth Hanssen, Jens C. Brüning, Marc Tittgemeyer

**Affiliations:** 1 Max-Planck-Institute for Metabolism Research, Cologne, Germany; 2 University of Cologne, Faculty of Medicine and University Hospital Cologne, Department of Neurology, Cologne, Germany; 3 Cologne Cluster of Excellence in Cellular Stress and Aging associated Disease (CECAD), Cologne, Germany; 4 Center for Endocrinology, Diabetes and Preventive Medicine (CEPD), University Hospital of Cologne, Cologne, Germany

**Keywords:** Neurophysiology, Endocrine system and metabolic diseases

## Abstract

Olfactory perception determines food selection behavior depending on energy homeostasis and nutritional status. The mechanisms, however, by which metabolic signals in turn regulate olfactory perception remain largely unclear. Given the evidence for direct insulin action on olfactory neurons, we tested olfactory performance (olfactory threshold, olfactory discrimination) in 36 subjects of normal- and overweight after administration of three different insulin doses (40 I.U., 100 I.U., 160 I.U.) or corresponding placebo volume in a within-subject design. Poor peripheral insulin sensitivity as quantified by HOMA-IR in baseline condition and increases in systemic insulin levels reactive to intranasal administration predicted poor olfactory performance. In contrast, intranasal insulin enhanced odor perception with a dose-dependent improvement of olfactory threshold. These findings indicate a new diametric impact of insulin on olfactory perception depending on peripheral or central availability.

## Introduction

The sense of smell impacts food selection behavior. The olfactory system does not only help to identify the chemical composition of food but also serves as an internal sensor for the nutritional status^1,2^. Recent observations that odor perception even directly impacts central nervous circuitries^3,4^ and peripheral metabolism^5^ have clearly emphasized the relevance of the olfactory system for energy homeostasis, but the underlying mechanisms are still poorly understood.

Olfactory neurons interact with central nervous circuitries regulating food intake and energy expenditure^6^. Interestingly, already the smell of hidden food stimuli activates AgRP and POMC neurons in the hypothalamus^3,4^ suggesting a strong influence of olfactory inputs on hypothalamic regulation of energy homeostasis. Olfactory sensitivity, in turn, is dynamically modulated by the nutritional status. Fasting enhances olfactory acuity, while satiation attenuates olfactory performance^7–11^. In long term, overconsumption of high fat food causes a loss of olfactory sensory neurons, impairs olfactory functioning and alters olfaction driven behavior^12,13^. However, it remains unclear how odor perception is modulated by global energy homeostasis.

Insulin is a key candidate for mediating this interaction. Secreted after food intake, blood insulin levels dynamically reflect body fuel availability. In fact, the highest density of central insulin receptors and the highest insulin concentration are found on the olfactory bulb^14–16^. The transport across the blood brain barrier is higher in this area compared to the rest of the brain^17^ suggesting direct modulation of olfactory signaling by insulin. Indeed, acute euglycaemic hyperinsulinaemia deteriorates olfactory performance^18^. A similar effect was observed after intracerebroventricular insulin application in rats^19^. Intranasal insulin application in humans, however, provided so far inconsistent results: While decreased olfactory performance was reported in normosmic subjects^20^, better olfactory sensitivity was demonstrated after intranasal insulin administration in anosmic subjects^21^ and hyposmic subjects^22^.

Here, we investigated the role of peripheral insulin sensitivity, intranasal insulin and reactive blood insulin changes after intranasal administration in the regulation of olfactory performance in humans. To consider variability in peripheral insulin sensitivity, we recruited a sample of healthy- and overweight subjects (BMI 20–30 kg/m^2^). Given the evidence that insulin sensitivity is a better indicator for the metabolic state than body weight^23,24^, and that the homeostasis model assessment of insulin resistance (HOMA-IR) is a good proxy for peripheral insulin sensitivity, we hypothesized that poor insulin sensitivity assessed by HOMA-IR will predict poor olfactory performance. Second, we explored the modulatory effect of central insulin action on olfactory perception through intranasal administration of different doses. Intranasal insulin applications are associated with reactive blood insulin changes^25^ that might in turn interact with olfactory perception and mask the intranasal insulin effects. Therefore, we hypothesized that correcting for reactive changes of blood insulin levels will reveal a dose-dependent modulation of olfactory perception by intranasal insulin with stronger effects of higher doses.

## Research Design and Methods

### Participants

This article provides findings obtained from a sample of 36 healthy weight and overweight male volunteers (BMI 24.98 ± 2.6 kg/m²), who underwent an intranasal intervention study to test for insulin effects on olfactory acuity. All participants gave written informed consent to participate in the experiment. The informed consent forms and all experimental protocols were approved by the local ethics committee of the Medical Faculty of the University of Cologne (Cologne, Germany). All methods were performed according to relevant guidelines and regulations.

The participants were recruited from a pre-existing database of volunteers maintained at the Max-Planck-Institute for Metabolism Research. We only employed male participants to exclude variations of hormonal effects on the olfactory performance during the menstrual cycle^26^. All participants were non-smokers without any history of neurological, psychiatric, gastrointestinal or eating disorders, and without any special diets or medical treatments. We had to exclude three subjects in the course of the data analysis: two subjects exceeded the cut-off value in a depression score (BDI > 13), and one subject showed an outlying variance of test performance across testing days (Cochran C test, C(7,34) = 0.14, p = 0.001). In total, 33 subjects (BMI: 24.87 ± 2.6 kg/m^2^, age: 25.6 ± 2.9 yrs, HOMA-IR: 1.68 ± 0.9) were included in the complete data analysis.

### Experimental design

The study was carried out in a single-blinded, placebo-controlled, crossover design (Supplementary Fig. 1). Each volunteer participated in seven experimental sessions. Two subsequent sessions were 4–21 days apart from each other and started around the same time of the day (either at 8:00 a.m. or 10:00 a.m.). On each testing day, participants arrived fasted with the last meal before 10 p.m. of the previous day.

On the first testing day the subjects performed the olfactory tests without any intervention to measure the baseline olfactory performance. On the next six testing days each participant received on different testing days either 40 units (0.4 ml), 100 units (1.0 ml), or 160 units (1.6 ml) of insulin (I.U.; Huminsulin® Normal KwikPen 100 I.U./ml; Lilly Germany GmbH) or 0.4, 1.0 or 1.6 ml of placebo (Saline KwikPen, Lilly Germany GmbH) in a counterbalanced order. As placebo, the vehicle solution with the same conservations agents was used and, hence, was indistinguishable by smell or sensation from insulin (Supplementary Fig. 4). To directly reach the central nervous system and circumvent the blood brain barrier the intervention was administered intranasally. Insulin and placebo were administered with a precision air pump (Aero Pump, Hochheim, Germany) alternating between both nostrils with an interval of one minute to allow sufficient time for absorption. Each puff of the air pump contained 0.1 ml of solution (equal to 10 I.U.). During the administration the volunteers were sitting upright in a separate room, observed by the test leader. On each testing day, an intravenous catheter was inserted into the left forearm vein and a blood sample was drawn in baseline condition as well as 10, 25, and 70 min post-intervention to identify the effect of intranasal insulin on glucose, insulin, c-peptide, and cortisol level.

Thirty minutes post-intervention two subtests of the “Sniffin’ Sticks” test battery (Burghart Instruments, Wedel, Germany) were used to assess the olfactory threshold and discrimination. The Sniffin’ Sticks test kit using odor dispensing pen-like devices is a standard approach to assess nasal chemosensory performance in research and clinical setting with a high test-retest reliability (0.61 for threshold, 0.54 for discrimination testing)^27^ and validity compared to other assessment batteries such as the “Cross-Cultural Smell Identification Test” and the “Connecticut Chemosensory Clinical Research Center Test”^27–29^. In particular, the olfactory threshold test, that assesses the concentration where half of the stimuli are detected while the other half remain undetectable, does not involve the identification of the exact odorant stimulus and is therefore unrelated to episodic and sematic memory and cognitive performance^30^.

To determine the olfactory threshold a single staircase, three alternative forced choice (3-AFC) procedure was used with 16 different dilutions of n-butanol (BUT)^27,28^. The olfactory threshold is defined as the lowest odor concentration that can be reliably detected^31^. More precisely, the test starts with the lowest BUT concentration that has to be identified among three presented pens (the other two pens are impregnated with a solvent). A wrong choice is followed by the next triplet of pens with increased BUT concentration until the subject identifies the correct pen. After a BUT pen with a certain concentration is correctly identified two times in a row the examiner continues with the triplet of pens, which includes the pen with the immediately lower BUT concentration. This is defined as the first turning point. After the next observed error (second turning point) a higher concentration is then presented again. The olfactory threshold detection score is determined by the mean of the last four turning points. The higher the olfactory detection score - called olfactory threshold performance in our results - the lower the olfactory threshold. For the discrimination test, subjects were presented 16 triplets of common everyday odorants like vanilla or cinnamon with the same concentration in a 3-AFC procedure in a counterbalanced order^27,31^. Here, two pens of each triplet contain the same odor, while the third pen is impregnated with a different smell with the same concentration. The subject has to identify the pen that differs from the other two. Olfactory discrimination is defined as the number of correct responses. See the Supplementary information for the data table on olfactory performance (Supplementary Data 2).

At the beginning of each testing day as well as before and after the olfactory testing subjects were asked to rate their hunger, satiety, tiredness and the feeling of a stuffy nose on a continuous 10 cm visual analogue scale (0 = “not hungry at all/not satiated at all/not tired at all”, and 10 = “extremely hungry/extremely satiated/extremely tired”). At the end of each testing day, subjects were further asked to rate how difficult they perceived the olfactory test on a visual analogue scale (0 = “extremely easy” and 10 = ”extremely difficult”). Additionally, on the first testing day subjects had to answer to a set of questionnaires comprising the Beck Depression Inventory (BDI-II)^32^ and the Baratt Impulsiveness Scale (BIS-1)^33^.

### Statistical analysis

The effect of insulin intervention on biochemical data, ratings and olfactory test results was assessed by linear mixed effect models using the R package ‘NLME’ 3.1^34^. Linear mixed effect models allowed to account for both within- and between-subjects sources of variance and offered an equivalent yet more versatile alternative to repeated measures ANOVA^35^. In addition to a general error term, all models included a random intercept for each participant to account for the repeated measures within participants (denoted “error ~1|participant” in Wilkinson notation). All other effects were modeled as fixed effects. In the description of each linear mixed effect model below we provide the characteristics of the factors tested. Main and interaction effects were both tested, but only effects that were significant at a critical p-value of 0.05 were reported. Post-hoc tests were corrected for multiple comparisons using the Dunnett correction (R ‘LSMEANS’ package^36^).

In this study, we investigated the olfactory performance after intranasal application of different doses of insulin and the corresponding placebo volumes (independent, manipulated variable). In addition, we assessed the relation between olfactory threshold and the pre-experimental variable insulin sensitivity (quantified by HOMA-IR) and the reactive variable blood insulin change after intranasal insulin application.

To investigate the relation between body weight, insulin sensitivity, and olfactory performance (both threshold performance and discrimination performance), we first performed a Pearson correlation analysis between BMI, average baseline HOMA-IR and average test performance across all seven testing days. To further control for the pharmacological manipulation, we implemented a linear mixed effect model assessing the effect of baseline HOMA-IR and condition (baseline, three different insulin interventions, three different placebo interventions) on olfactory performance (olfactory threshold or olfactory discrimination). Accordingly, we tested the effect of BMI and conditions on olfactory performance. The homeostasis model assessment of insulin resistance (HOMA-IR)^37,38^ is a proxy for insulin sensitivity and calculated as

$$\text{HOMA} \mbox{-} \text{IR}=\frac{{\rm{fasting}}\,{\rm{serum}}\,{\rm{glucose}}\,[{\rm{mg}}/dl]\times {\rm{fasting}}\,{\rm{serum}}\,{\rm{insulin}}\,[{\rm{mU}}/{\rm{l}}]}{405}.$$

Higher values indicate a lower degree of insulin sensitivity. As the HOMA-IR was initially not normally distributed, we applied a logarithmic transformation prior to analysis.

In the following we only used linear mixed effect models including the random effect for the factor participant.

To start with, we tested the effect of intervention (categorical factor, 2 levels: insulin, placebo) and test modality (categorical factor, 2 levels: olfactory threshold, olfactory discrimination) on olfactory performance. We beforehand z-scored test-performances to allow the comparison of olfactory performances across subjects and test modalities (olfactory threshold and olfactory discrimination).

Next, to assess the dose dependent modulation of blood insulin and blood glucose by intranasal insulin we analyzed the effect of intervention dose (categorical factor, 4 levels: 40 I.U., 100 I.U., 160 I.U, average placebo) and time (categorical factor, 4 levels: baseline, 10, 25, 70 min post intervention) on either blood insulin or blood glucose.

We then tested whether reactive blood insulin changes after intranasal insulin administration modulated olfactory threshold performance by setting up a model testing the effect of intranasal insulin dose (categorical factor, 3 levels: 40 I.U., 100 I.U. and 160 I.U.) and blood insulin level prior to olfactory testing in the insulin compared to the placebo condition (Δ blood insulin_(insulin–placebo)_, continuous factor) on the olfactory threshold performance in the insulin compared to placebo condition (Δ olfactory threshold performance_(insulin-placebo)_, continuous factor).

Finally, we corrected the olfactory threshold values by regressing out the blood insulin level prior to olfactory assessment (25 min post intervention) and consequently analyzed the effect of intervention (categorical factor, 2 levels: placebo, insulin) and insulin dose (categorical factor, 3 levels: 40 I.U, 100 I.U., 160 I.U.) on the corrected olfactory threshold performance.

We controlled for differences in internal states by testing the effect of time (baseline, 10 min, 25 min and 70 min) and intervention dose on the subjects’ ratings of satiety, hunger, and tiredness.

The order of the testing days was counterbalanced by pseudo-randomly assigning the participants to one of five groups, each group being associated with a different carefully predetermined order of the six interventions. To analyze a potential order effect we used a linear mixed model analyzing the effect of group (categorical factor, 5 levels) on either olfactory threshold or olfactory discrimination.

All data are reported as the mean ± SEM.

## Results

To investigate the effect of intranasal insulin application on olfactory performance, we tested 33 male volunteers in a weight range between normal- and overweight.

**Figure 1:**
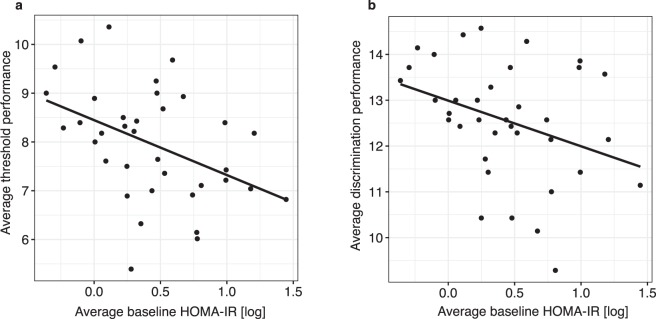
Baseline insulin sensitivity and olfactory performance: Average baseline HOMA-IR across all seven testing days was correlated with both (**a**) average olfactory threshold (*r(31)* = −*0*.*455*, *p* = *0*.*008*) and (**b**) average olfactory discrimination (*r(31)* = −*0*.*352*, *p* = *0*.*04*) performance across testing days. (Baseline HOMA-IR was converted by logarithmic transformation).

First, we investigated the effect of baseline insulin sensitivity on olfactory performance. While there was no correlation with BMI, average performance of both olfactory threshold (*r*(*31*) = −*0*.*455*, *p* = *0*.*008*) as well as odor discrimination (*r(31)* = −*0*.*352*, *p* = *0*.*04*) were correlated with average HOMA-IR at baseline level. Moreover, to account for the different conditions across testing days (baseline, three different insulin and placebo interventions) we performed linear mixed effect models testing the effect of baseline HOMA-IR and conditions on olfactory performance (olfactory threshold/olfactory discrimination). In line with the correlation analysis, beside the main effect of condition (*F(6*,*185)* = *2*.*67*, *p* < *0*.*016*) we found a main effect of baseline HOMA-IR (*F(1*,*185)* = *6*.*86*, *p* = *0*.*009*) on olfactory threshold. For olfactory discrimination, there was a main effect of condition (*F(6*,*185)* = *2*.*83*, *p* = *0*.*011*) and a statistical trend for the main effect of baseline HOMA-IR HOMA-IR (*F(1*,*185)* = *2*.*28*, *p* = *0*.*100*). In contrast, testing the effect of BMI and conditions on olfactory performance (olfactory threshold/olfactory discrimination) we could not find a main effect of BMI. In other words, higher HOMA-IR, i.e. lower systemic insulin sensitivity, rather than increased body weight *per se* was associated with poor performance in olfactory tasks (Fig. 1).

**Figure 2:**
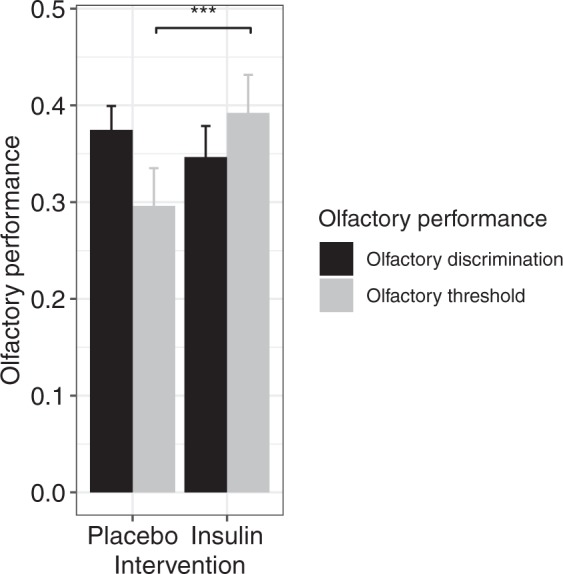
Improved olfactory performance after intranasal insulin: The effect of intranasal insulin (across all intervention doses) was specific to olfactory modality. It improved olfactory threshold, while olfactory discrimination remained unchanged. (Means ± SEM, ***p ≤ 0.001, **p ≤ 0.01, *p ≤ 0.05, the values are z-transformed).

Next, we explored the effect of local intranasal insulin application on olfactory functioning. Surprisingly, intranasal insulin improved the sense of smell (Fig. 2). This effect was specific to olfactory threshold performance, while the discrimination of olfactory stimuli remained unchanged after intranasal insulin application *(main effect of intervention: F(1*,*360)* = *4*.*970*, *p* = *0*.*0264*, *interaction effect intervention × test type: F(1*,*360)* = *6*.*031*, *p* = *0*.*01*).

To further identify possible dose dependent effects of intranasally applied insulin on olfactory performance, we compared the olfactory performance after application of 40 I.U., 100 I.U. and 160 I.U. intranasal insulin.

**Figure 3:**
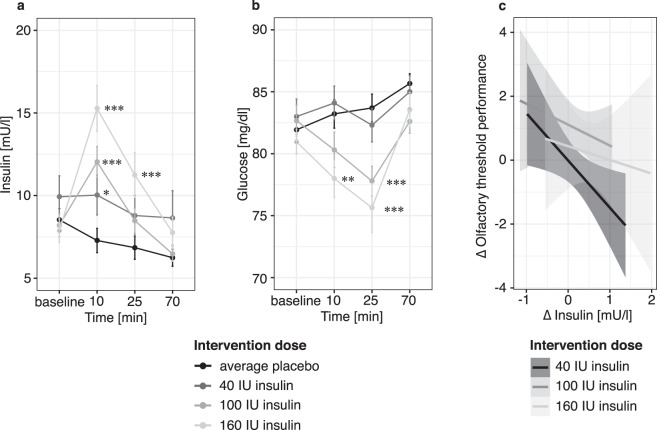
Effects of systemic insulin on olfactory threshold: Intranasal insulin administration caused a time- and dose-dependent modulation of (**a**) systemic insulin and (**b**) glucose level. (Means ± SEM, ***p ≤ 0.001, **p ≤ 0.01, *p ≤ 0.05). (**c**) Both systemic and intranasal insulin had an effect on olfactory threshold: Higher systemic Δ insulin predicted poor Δ olfactory threshold performance. (Δ insulin = blood insulin level 25 min post intervention in insulin compared to placebo condition, Δ Olfactory threshold performance = threshold test in insulin compared to placebo condition).

**Figure 4:**
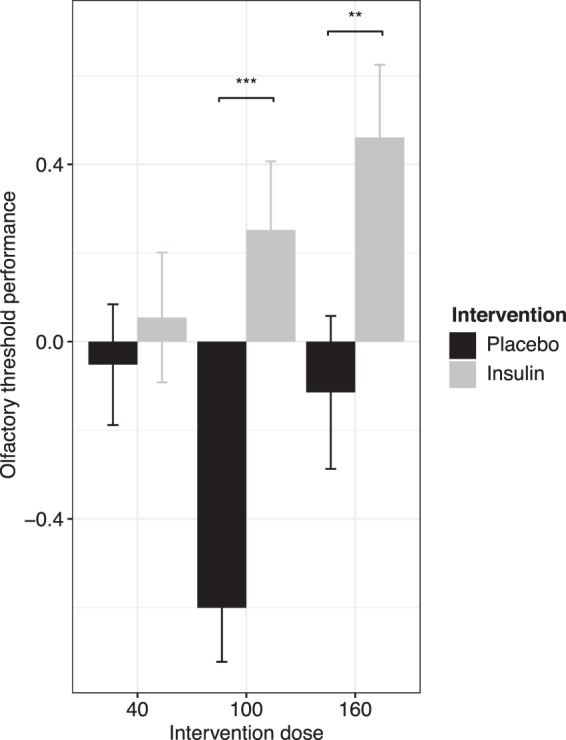
Improved olfactory threshold after higher doses of intranasal insulin: Intranasal insulin showed a dose dependent effect on olfactory threshold performance. We found an interaction of intervention and intervention dose. 100 I.U. and 160 I.U. intranasal insulin improved olfactory threshold compared to the corresponding placebo condition (Means ± SEM, ***p ≤ 0.001, **p ≤ 0.01, the values are z-scored).

Before going further, it is important to note that in line with previous reports intranasal insulin application induced reactive blood insulin changes. It increased blood insulin (*no significant main effect*, *interaction effect of time × dose: F(9*,*380)* = *6*.*595*, *p* < *0*.*0001*) and reduced blood glucose (*no significant main effect*, *interaction effect of time × dose: F(9*,*380)* = *16*.*044*, *p* < *0*.*0001*) level in a time- and dose-dependent manner (Fig. 3a,b). This observation raised the question whether the shift in blood insulin level prior to olfactory testing in the insulin compared to the placebo condition (Δ blood insulin, insulin - placebo) affected the olfactory performance. Hence, we assessed the effect of Δ blood insulin on the olfactory threshold performance. Indeed, we found that both blood insulin and intranasal insulin intervention dose had an effect on olfactory threshold *(main effect of blood insulin: F(2*,*61)* = *4*.*772*, *p* = *0*.*03)*, *main effect of intervention dose: F(2*,*61)* = *5*.*081*, *p* = *0*.*009)*. Higher Δ blood insulin predicted poorer olfactory threshold performance (Fig. 3c). To better delineate the effect of local insulin on olfactory threshold performance, we then tested the effect of intranasal insulin after correcting for (i.e. regressing out) the effect corresponding to the blood insulin level prior to olfactory testing (25 minutes post-intervention). This analysis showed a main effect of intervention *(F(1*,*160)* = *17*.*351*, *p* = *0*.*001)* and revealed an interaction of intervention and dose *(F(2*,*160)* = *3*.*144*, *p* = *0*.*04)*. Strikingly, post-hoc tests showed that 100 I.U. *(p* = *0*.*0001*, *t* = −*4*.*0)* and 160 I.U. *(p* = *0*.*0076*, *t* = −*2*.*7)* of intranasal insulin induced a strong improvement of olfactory threshold performance compared to the corresponding placebo dose *(*Fig. 4*)*. In other words, while higher systemic insulin levels predicted poorer olfactory performance, higher intranasal insulin doses improved olfactory threshold.

To control for differences in internal states, we instructed the participants to rate their hunger, satiety, and tiredness at baseline and immediately before and after olfactory testing on each day. Interestingly, hunger (*main effect of time: F(2*,*254)* = *16*.*24*, *p* < *0*.*0001*) and tiredness (*main effect of time: F(2*,*254)* = *7*.*50*, *p* = *0*.*0007*) increased and satiety dropped (*main effect of time: F(2*,*254)* = *12*.*63*, *p* < *0*.*0001*) over time, but we could not find an effect of intranasal insulin application on these parameters (Supplementary Fig. 3). In addition, we tested for a potential order effect, as all participants performed olfactory tests for seven times. We could not find an effect of order neither on olfactory threshold *(F(1*,*164)* = *1*.*54*, *p* = *0*.*22)* nor on olfactory discrimination *(F(1*,*164)* = *0*.*98*, *p* = *0*.*32)* performance.

## Discussion

Recent findings indicate a strong impact of metabolic state and body weight on olfactory perception, however very little information has been gathered on the underlying mechanisms^1,39–41^. Given the evidence for direct insulin action on neuronal activity^42,43^ and cell regeneration in the olfactory system^44^, we systematically investigated the modulatory effects of intranasal insulin on olfactory performance. In addition we characterized the role of insulin-sensitivity and reactive blood insulin changes after intranasal insulin application on olfactory threshold. Our findings suggest that systemic and local insulin modulate smell perception in opposing direction.

The olfactory bulb is considered as an internal sensor for the nutritional status. Besides other gastrointestinal signals, insulin has been shown to directly act on olfactory neurons^43,45^. This is particularly interesting, since systemic insulin levels dynamically reflect body fuel availability. We demonstrate that a transient increase in blood insulin modulated olfactory performance. Consistent with previous hyperinsulinemic clamp data^18^, higher blood insulin predicted poorer olfactory performance. These findings extend to the assumption that changes of olfactory sensitivity are induced by satiety signals^18,19,31^.

With increasing body weight insulin homeostasis is impaired, leading to hyperinsulinemia and insulin resistance. Previous studies reported olfactory dysfunction in morbidly obese subjects^39,46^ and diabetes^47^. Odor sensitivity has been found to decrease with increasing BMI^40,41^. We discovered that not body weight itself but blood insulin level and insulin sensitivity determine olfactory threshold. Higher baseline HOMA-IR or circulating baseline insulin levels were correlated with poorer olfactory performances. Future studies need to clarify whether normalization of peripheral insulin sensitivity leads to a restored olfactory acuity. Interestingly, our pilot assessment of nine morbidly obese subjects (BMI: 42.8 ± 5.24 kg/m^2^, Supplementary Data 1) undergoing a weight loss program indicated an improvement of olfactory acuity with decreasing peripheral insulin levels (Supplementary Fig. 7).

Surprisingly, intranasal insulin had a contrary effect. We found enhanced olfactory performance after intranasal insulin administration. The olfactory threshold was selectively improved, while odor discrimination remained unchanged. Interestingly, this effect was dose-dependent. We discovered best improvement after 100 I.U. and 160 I.U. intranasal insulin.

At the first glance, these results seem contradictory to the assumption that insulin signals satiety and reduces odor sensitivity. However, it is established that centrally applied insulin often results in opposite effects compared to peripheral application suggesting a counter-regulatory mechanism^48^. Blood borne insulin crosses the blood brain barrier (BBB) through a saturable transport mechanism^17,49^. Blood and olfactory bulb insulin levels are elevated in sated compared to fasted state, but these levels are not correlated and the ratio changes with increasing blood insulin level^19^. Intranasal application bypassing the BBB results in unphysiological elevation of central insulin levels^50^ that may evoke opposing effects on olfactory sensitivity. This is further supported by the fact that we found a stronger improvement for high intervention doses. However, it is important to note that intranasal insulin does not specifically target the olfactory bulb but may also cause activity changes in other components of the olfactory system such as the olfactory epithelium and the olfactory cortex.

Intranasal insulin application is widely considered as the standard procedure to mimic central nervous insulin actions in humans. However, in line with previous literature^25^ we demonstrate a profound dose-dependent increase of blood insulin with subsequent reductions in glucose concentration. Previous human and animal work suggested several mechanisms underlying these reactive changes such as spill over effects, vagally transmitted insulin secretion^51^, suppression of endogenous glucose production^52^ or even classical conditioning^53^. However, the short interval between intranasal insulin administration and peripheral insulin rise in our study suggest spill-over effects. Our results clearly indicate the relevance of controlling for fasting state, blood insulin level and peripheral insulin sensitivity when testing the effects of intranasal insulin. Inconsistencies in these parameters might explain the contradictory reports on intranasal insulin effects on olfactory threshold so far^20,21,45^.

In conclusion, our findings indicate opposing effects of central and blood insulin on olfactory threshold performance. While intranasal insulin dose-dependently improved olfactory threshold, increasing blood insulin levels and poor insulin sensitivity predicted poor olfactory performance. Given the evidence, that olfactory sensitivity directly impacts central homeostatic regulation^3,4^ and peripheral metabolism^5^, being able to modulate olfactory acuity through intranasal insulin application represents a highly attractive potential for clinical application.

## Supplementary information

Supplementary Information

Dataset 2

## Data Availability

The data on olfactory performance and blood parameters are included in the Supplementary Information files. Further datasets generated during and/or analyzed during the current study are available from the corresponding author on reasonable request.
