# Supplementary material for: The role of insulin sensitivity and intranasally applied insulin on olfactory perception
Source: Sci Rep. 2019 May 10;9:7222. doi: 10.1038/s41598-019-43693-7 (PMC6510903; doi:10.1038/s41598-019-43693-7)
Supplement: Supplementary file 1 — Supplementary Information [file 41598_2019_43693_MOESM1_ESM.pdf]

Supplementary Data: The role of insulin sensitivity and intranasally applied insulin on olfactory performance

Sharmili Edwin Thanarajah[1,2], Vera Hoffstall[1], Lionel Rigoux[1], Ruth Hanssen[1,4], Jens C. Bruening[1,3,4], Marc Tittgemeyer[1,5]

[1]Max-Planck-Institute for Metabolism Research, Cologne, Germany

[2] University of Cologne, Faculty of Medicine and University Hospital Cologne, Department of Neurology

[3]Cologne Cluster of Excellence in Cellular Stress and Aging associated Disease (CECAD), Cologne, Germany

[4]Center for Endocrinology, Diabetes and Preventive Medicine (CEPD), University Hospital of Cologne, Cologne, Germany

[5]Modern Diet and Physiology Research Center, USA

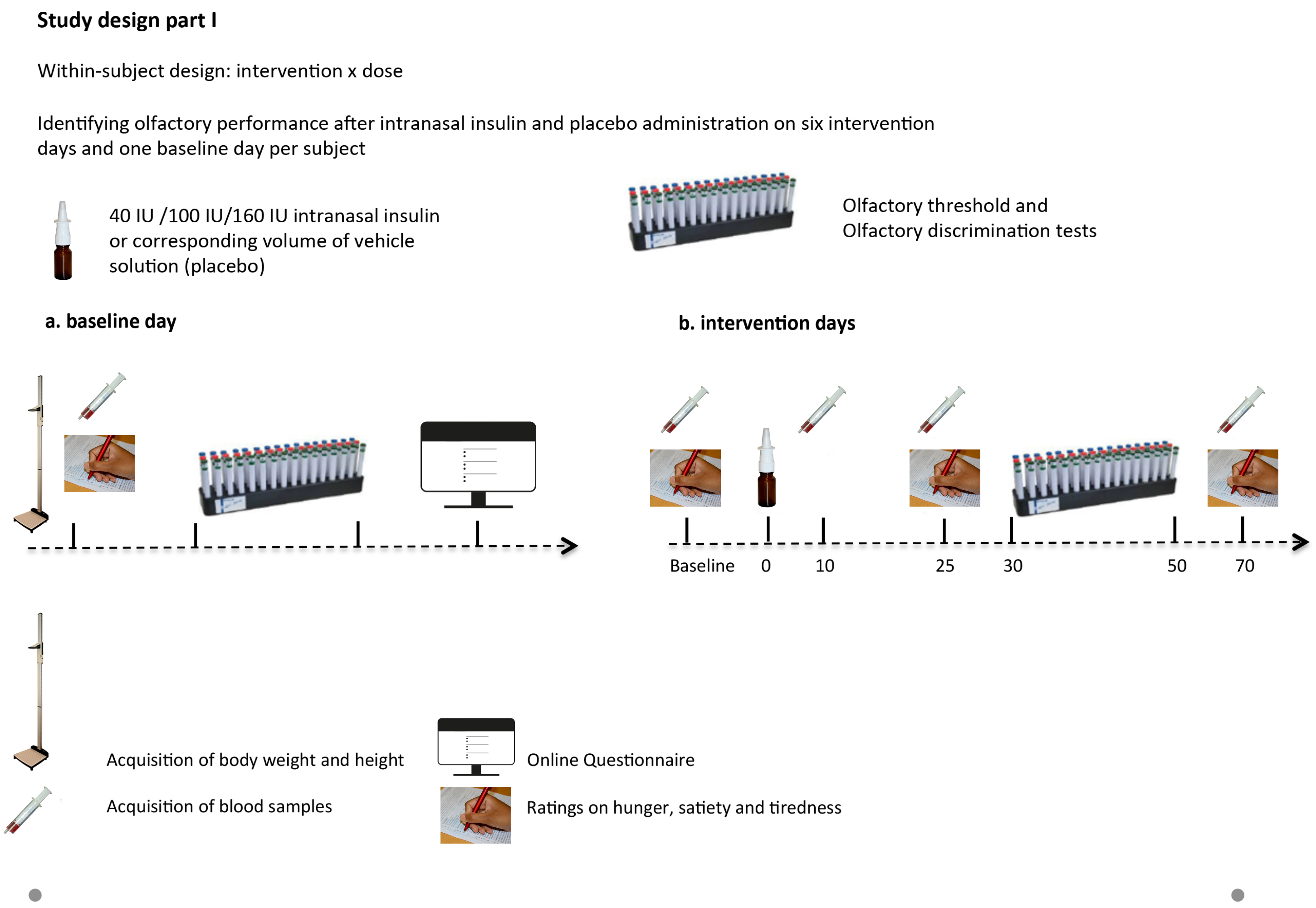
Study design part I
Within-subject design: intervention x dose
Identifying olfactory performance after intranasal insulin and placebo administration on six intervention days and one baseline day per subject
40 IU /100 IU/160 IU intranasal insulin or corresponding volume of vehicle solution (placebo)
Olfactory threshold and Olfactory discrimination tests
a. baseline day
b. intervention days
Baseline
0
10
25
30
50
70
Acquisition of body weight and height
Acquisition of blood samples
Online Questionnaire
Ratings on hunger, satiety and tiredness

Supplementary Figure 1: Study design. Placebo-controlled, randomized, crossover design: All volunteers participated on seven testing days after an overnight fast. **a.) Baseline testing day:** After blood sampling and acquiring data on body weight, height and internal state, we tested olfactory threshold and discrimination. Subsequently the participants completed questionnaires on depression and impulsivity. **b.) Intervention days:** After internal state ratings and blood sampling in baseline condition, either 40 I.U., 100 I.U. or 160 I.U. insulin or the corresponding placebo volume were intranasally administered in a randomized order. Blood samples and ratings on the internal state were acquired 10, 25 and 70 minutes post-intervention. We performed standardized olfactory threshold and discrimination testing 30 minutes after the intervention. Before and after olfactory testing the participants rated on hunger, satiety and tiredness.

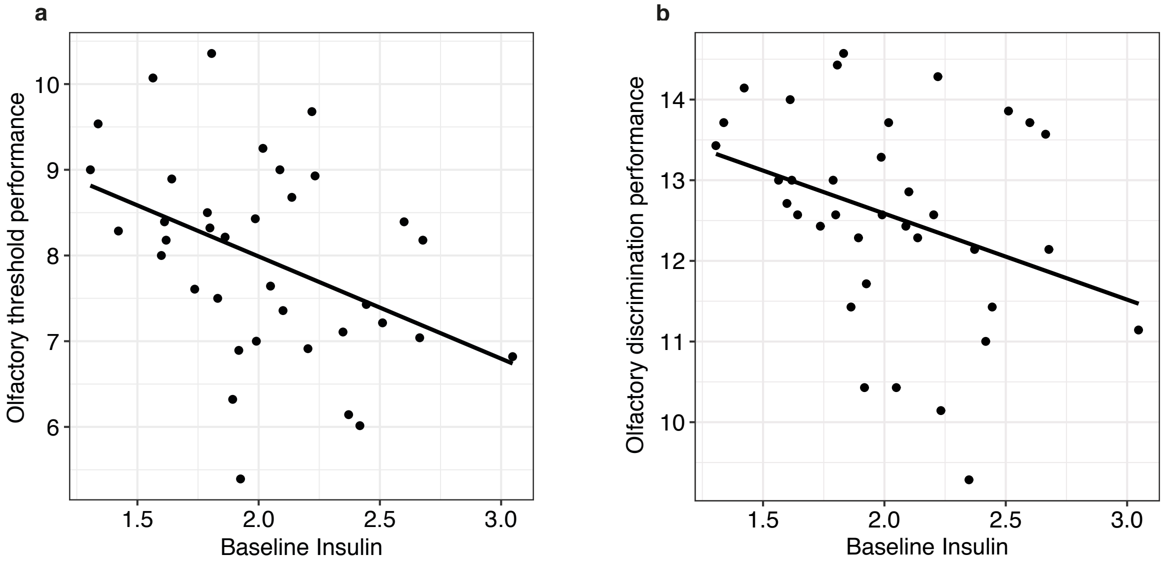

Supplementary Figure 2: Baseline metabolic state and olfactory performance. Baseline insulin level predicted both *a.*) olfactory threshold ($r(31)=-0.44, p=0.01$) and *b.*) olfactory discrimination ($r(31)=-0.35, p=0.04$).

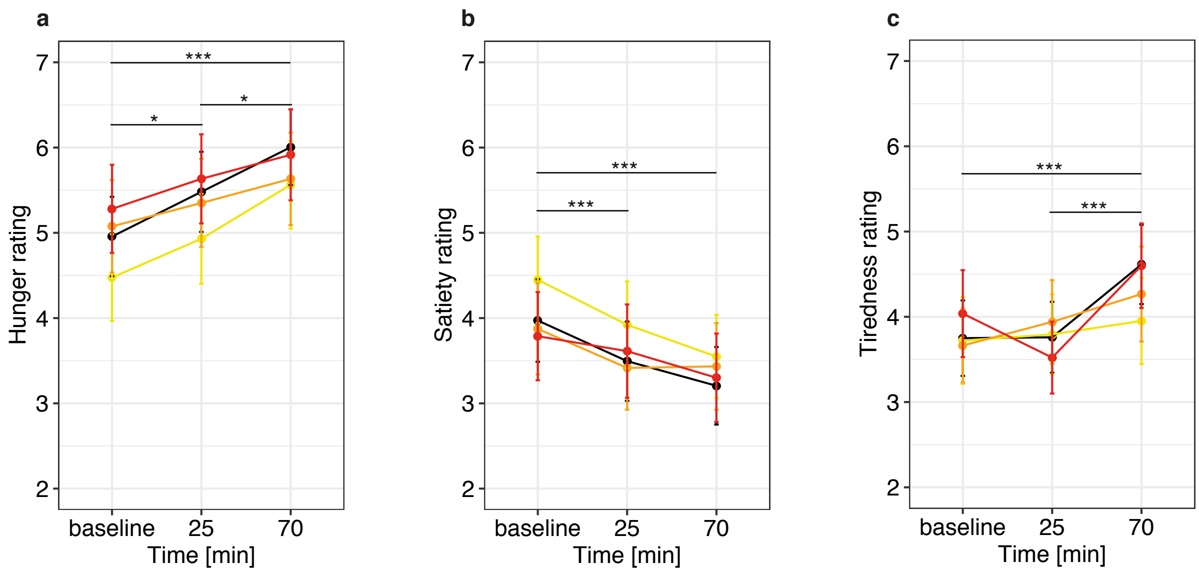

Supplementary Figure 3: Intranasal insulin does not modulate hunger and satiety. . *a.*) Hunger, *b*) satiety and *c.*) tiredness changed across time, but were not modulated by insulin intervention. (Means ± SEM, *** $p \leq 0.001$, **$p \leq 0.01$, * $p \leq 0.05$)

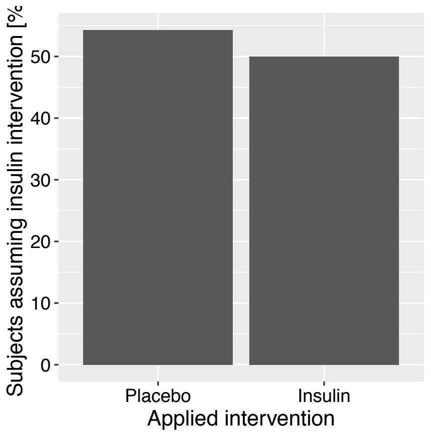

Supplementary Figure 4: Intranasal insulin and placebo were indistinguishable: In a separate cohort 17 lean (BMI: 22.9 kg/m^2 ± 1.5, age: 27.0 yrs ± 4.0) and 19 overweight subjects (7.2 kg/m^2 ± 1.5, age: 30.0 yrs ± 4.9) received intranasal administration of either 100 I.U. insulin or the corresponding placebo (vehicle solution) volume on two different days in a randomized order. The participants were asked to rate whether they received insulin or placebo. On both days approximately 50 % of the subjects assumed an intranasal insulin application.

Supplementary Data 1: In a pilot assessment 10 obese individuals (BMI: 42.52 ± 5.02 kg/m^2, age: 49.29 ± 10.74, HOMA-IR 5.05 ± 2.4) of both genders (6 female) who participated in a weight loss program were included in the study. All participants were non-smokers without any history of neurological disorders or diabetes. We had to exclude one subject in the course of the data analysis, due to a flu with nasal congestion on the second testing day. In total, 9 subjects (BMI: 42.8 ± 5.24 kg/m^2, age: 49.2 ± 11.4 yrs, HOMA-IR 5.04 ± 2.4) were included in the complete data analysis.

We tested the effect of weight loss on olfactory performance (*Supplementary Figure 5*); no intranasal insulin was administered. The participants underwent the OPTIFAST52-Programm at the Center for Endocrinology, Diabetology, and Preventive Medicine at the University hospital of Cologne and replaced their normal diet by a formula (OPTIFAST 800) diet consisting of approximately 800 kcal/day for 12 weeks. Each volunteer participated on two experimental sessions around the same time of the day (either at 8:00 a.m. or 10:00 a.m.). The first session was conducted at baseline, the second session took place after completing a 12 weeks weight loss program. On each testing day, participants arrived fasted with the last meal before 10 p.m. of the previous day. At the beginning of each testing day a blood sample was drawn to measure insulin and glucose level and the body weight and height was acquired. The olfactory threshold and discrimination were assessed using "Sniffing' Sticks" as described above. Before and after olfactory testing subjects were asked to rate their hunger, satiety, tiredness and the feeling of having a stuffy nose on a continuous 10 cm visual analogue scale (0 = "not hungry/satiated/tired at all", and 10 = "extremely hungry/satiated/tired").

After the dietary intervention we found reduced body weight and improved systemic insulin sensitivity (*Supplementary Figure 6*). We tested the effect of change in BMI (*Δ* BMI) and basal blood insulin (*Δ* insulin) on olfactory threshold performance. We found that stronger reduction in baseline insulin level predicted better improvement in olfactory acuity (main effect of *Δ* insulin, $F(1,6)=6.42$, $p=0.04$, *Supplementary Figure 7*).

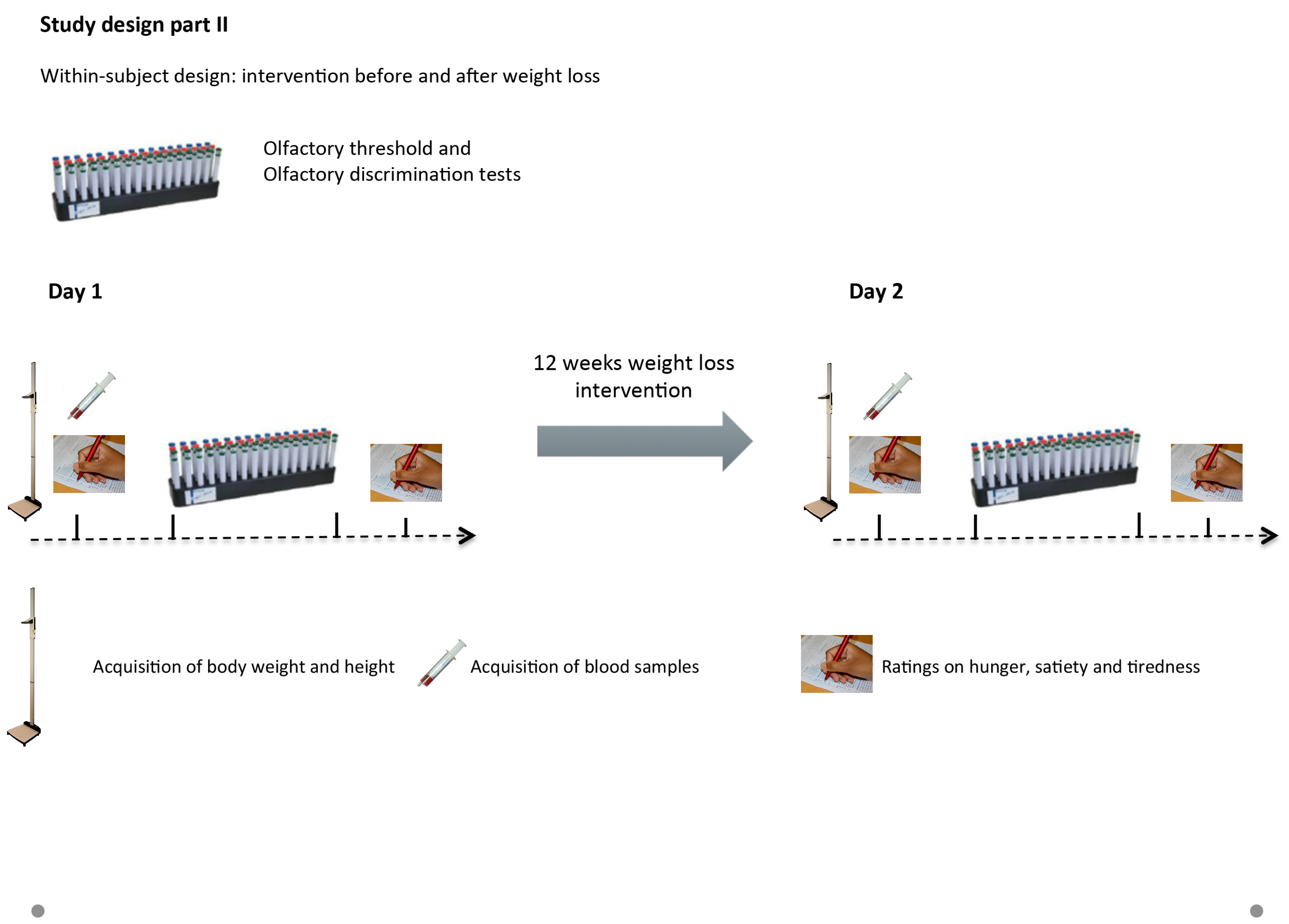
Study design part II
Within-subject design: intervention before and after weight loss
Olfactory threshold and
Olfactory discrimination tests
Day 1
12 weeks weight loss
intervention
Day 2
Acquisition of body weight and height
Acquisition of blood samples
Ratings on hunger, satiety and tiredness

Supplementary Figure 5: Study design part II. Subjects were tested before and after a 12-weeks low caloric diet. On both testing days, subjects arrived after an overnight fast. After a blood draw and acquisition of body weight and height subjects underwent standardized olfactory discrimination and olfactory threshold tests. Ratings on hunger, satiety and tiredness were acquired before and after olfactory testing.

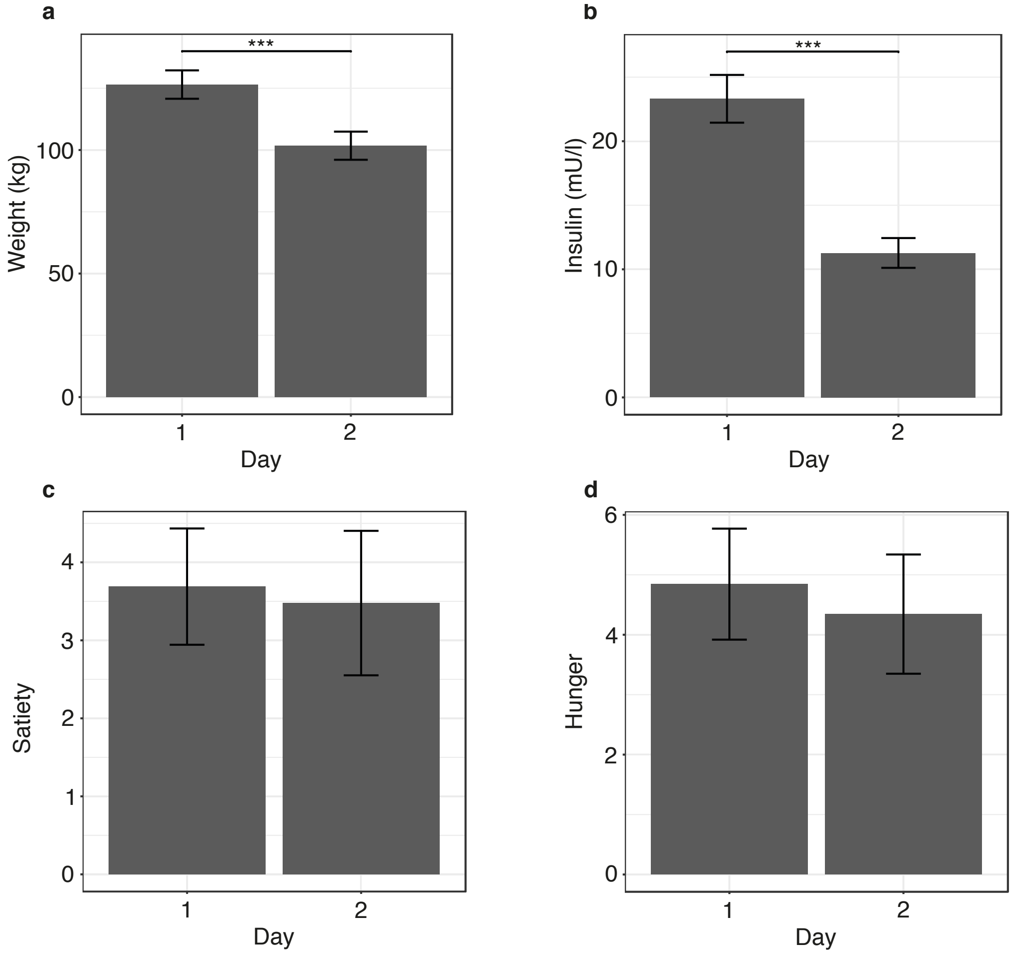

Supplementary Figure 6: Changes after 12 weeks weight loss program: After the 12 weeks weight loss program, subjects presented a drop of *a.*) body weight ($p < 0.001$, t=11.6) and *b.*) baseline insulin level ($p < 0.001$, t=9.3). *c.*) Satiety and *d.*) hunger ratings in baseline condition did not change after the formula diet.

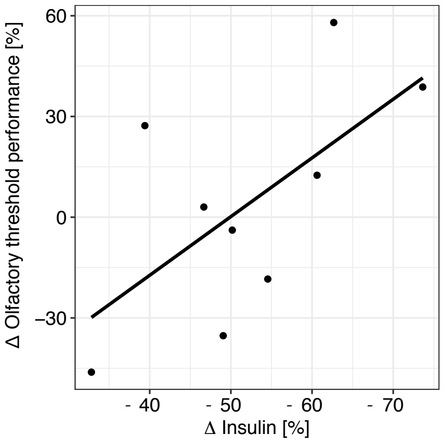

Supplementary Figure 7: Normalization of insulin levels after weight loss restored olfactory acuity: Greater reduction in blood insulin level (*Δ* insulin) predicted better olfactory threshold performance controlling for the change in BMI ($F(1,6)=6.42$, $p=0.04$).

Supplementary Data 2: Full data table with olfactory threshold and olfactory discrimination performances and the intervention applied.
